# Coexistence of Cervical Leiomyosarcoma and Gastric-Type Adenocarcinoma In Situ with Extensive Extension to the Endometrium and Fallopian Tube

**DOI:** 10.1155/2018/5848629

**Published:** 2018-02-18

**Authors:** Ayako Ura, Kanako Ogura, Asumi Sakaguchi, Hiroko Onagi, Daiki Ogishima, Yayoi Sugimori, Kensuke Hamamura, Masaharu Fukunaga, Toshiharu Matsumoto

**Affiliations:** ^1^Department of Diagnostic Pathology, Juntendo University Nerima Hospital, Tokyo, Japan; ^2^Department of Gynecology, Juntendo University Nerima Hospital, Tokyo, Japan; ^3^Department of Diagnostic Pathology, Shin-Yurigaoka General Hospital, Kanagawa, Japan

## Abstract

Cervical leiomyosarcoma is known to be rare from the previous reviews of a large number of malignant cervical tumors. The patient was a 66-year-old woman with irregular vaginal bleeding. She underwent modified radical hysterectomy and bilateral salpingooophorectomy. Histopathologically, we diagnosed the coexistence of uterine cervical leiomyosarcoma and cervical gastric-type adenocarcinoma in situ with endometrial lesions that had continuous and skip patterns and fallopian tubal lesions with a partial lesion. To the best of our knowledge, cases of synchronous leiomyosarcoma and cancers have not often been reported; only two cases of synchronous cervical leiomyosarcoma and cervical squamous cell carcinoma have been published. This case is the first presentation of coincidental primary cervical leiomyosarcoma and cervical gastric-type adenocarcinoma in situ. Additionally, we considered cervical gastric-type adenocarcinoma in situ with continuous lesions on the endometrium and skip lesions on the left fallopian tube.

## 1. Introduction

Cervical sarcoma is a rare neoplasm; Wright et al. reported that among 1583 patients with cervical malignancies, eight cervical sarcomas were identified, and only one patient had leiomyosarcoma [1]. Thereafter, Khosla et al. reported similar findings in 1804 patients with cervical malignancies; eight cervical sarcomas were identified, and three patients had leiomyosarcoma [2]. Cervical leiomyosarcoma is extremely rare; only six cases have been reported, only two of which were synchronous endocervical leiomyosarcoma and endocervical squamous cell carcinoma [3–7].

To the best of our knowledge, synchronous occurrence of leiomyosarcoma and adenocarcinoma in the cervix has not been reported.

Here, we reported the first case with synchronous occurrence of leiomyosarcoma and adenocarcinoma in situ in the cervix. Moreover, in our case, synchronous occurrence of intraepithelial carcinoma was observed in the endometrium and fallopian tube.

## 2. Case Presentation

### 2.1. Clinical Course

**Figure 1:**
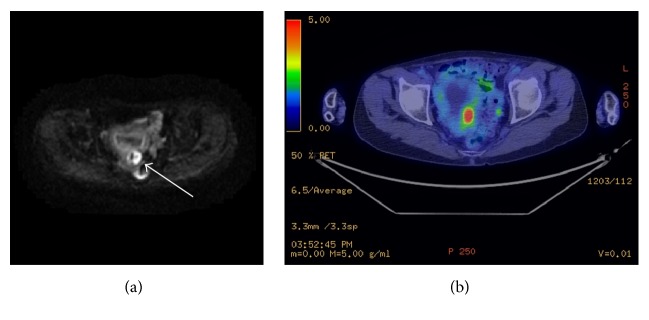
(a) Diffusion weighted magnetic resonance imaging. The abnormal signal with a diameter of approximately 2 cm on the left sidewall of the uterine corpus (at indicated by arrow). (b) FDG-PET. Abnormal uptake in the tumor had a maximal standardized uptake value (SUV (max)) of 7.79.

The patient, a 66-year-old gravida one-para one woman, was referred to Juntendo University Nerima Hospital with irregular vaginal bleeding and watery vaginal discharge. The hydrometra, with a diameter of approximately 14 × 11 cm by ultrasound examination, was found. Diffusion weighted magnetic resonance imaging revealed an abnormal mass with a diameter of approximately 2 cm on the left sidewall of the uterine corpus without any lymph node enlargement (Figure 1(a)). Fluorodeoxyglucose-positron emission tomography showed abnormal uptake in the mass with a maximal standardized uptake value (SUV, max) of 7.79 (Figure 1(b)). Serum tumor markers, including CEA, CA19-9, and CA125, were within normal limits.

Histological examination of the endometrial biopsy established the diagnosis of leiomyosarcoma and atypical endometrial epithelial lesion. A modified radical hysterectomy and bilateral salpingooophorectomy were performed. After the surgery, the patient was not treated with additional treatments. She had no recurrence within seven months after the operation.

### 2.2. Pathological Finding of Resected Tissues

**Figure 2:**
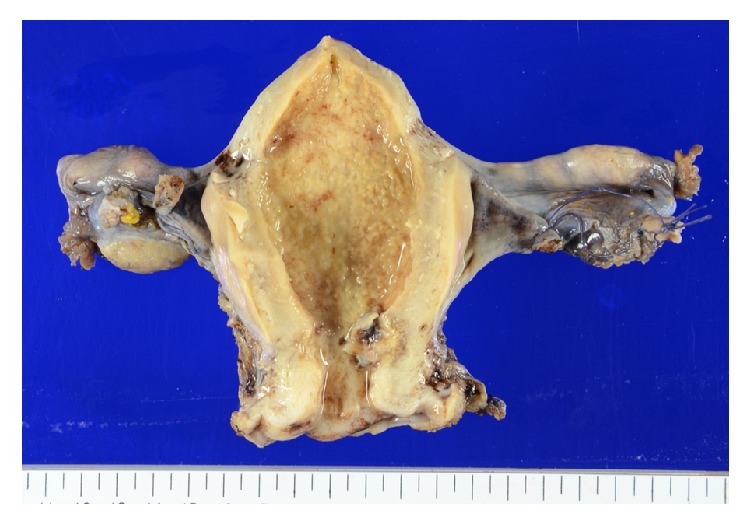
The specimen from the operation. There was a 1.5 cm brownish mass with an unclear border with the stroma on the uterine corpus side of the cervix.

In the uterus, a 1.5 cm sized brownish mass was found in the cervix near the corpus; it was located in the stroma with an irregular border. The lumen of the corpus was expanded, and the mucosa was diffusely irregular. There was no nodule or tumorous mass in the corpus. There were no abnormal findings in the bilateral adnexa (Figure 2).

**Figure 3:**
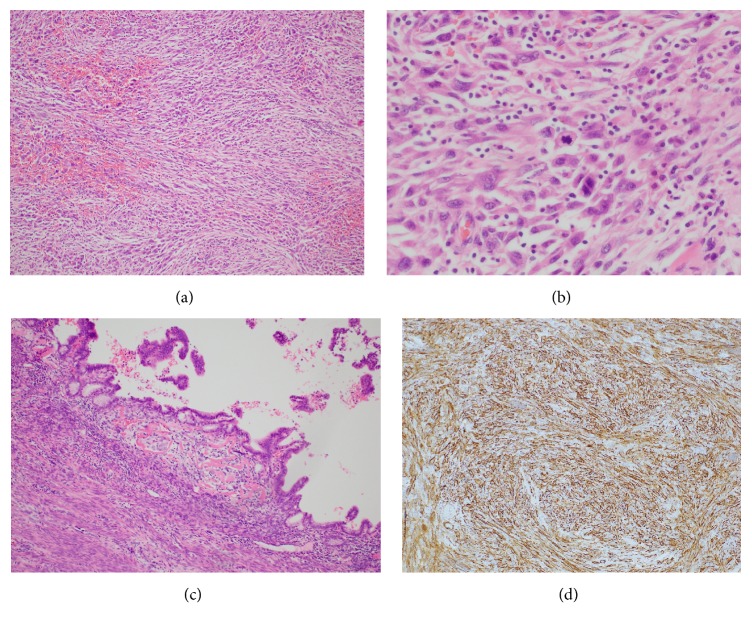
Endocervical tumor. (a) H-E stain. Fascicular and interlacing proliferation of spindle cells with hemorrhage. (b) H-E stain. Spindle cells had high-grade atypia and active mitosis. (c) H-E stain. Atypical cells were infiltrating just beneath the mucosa. (d) Immunohistochemical stain of H-caldesmon. Tumor cells were diffuse positive.

Histologically, the cervical mass was highly cellular and consisted of densely packed spindle-shaped to oval-shaped cells with high-grade atypia with a fascicular patterned arrangement and occasional lymphocytic infiltration. It infiltrated beneath the mucosa (Figures 3(a) and 3(c)). High mitotic activity and focal necrosis with hemorrhage were also noted in the mass (Figure 3(b)). Immunohistochemically, the spindle and oval cells were positive for vimentin, SMA, and H-caldesmon (Figure 3(d)) and were negative for epithelial markers and ALK (Table 1). From the histological findings and immunohistochemical results, the mass was finally diagnosed as leiomyosarcoma.

**Figure 4:**
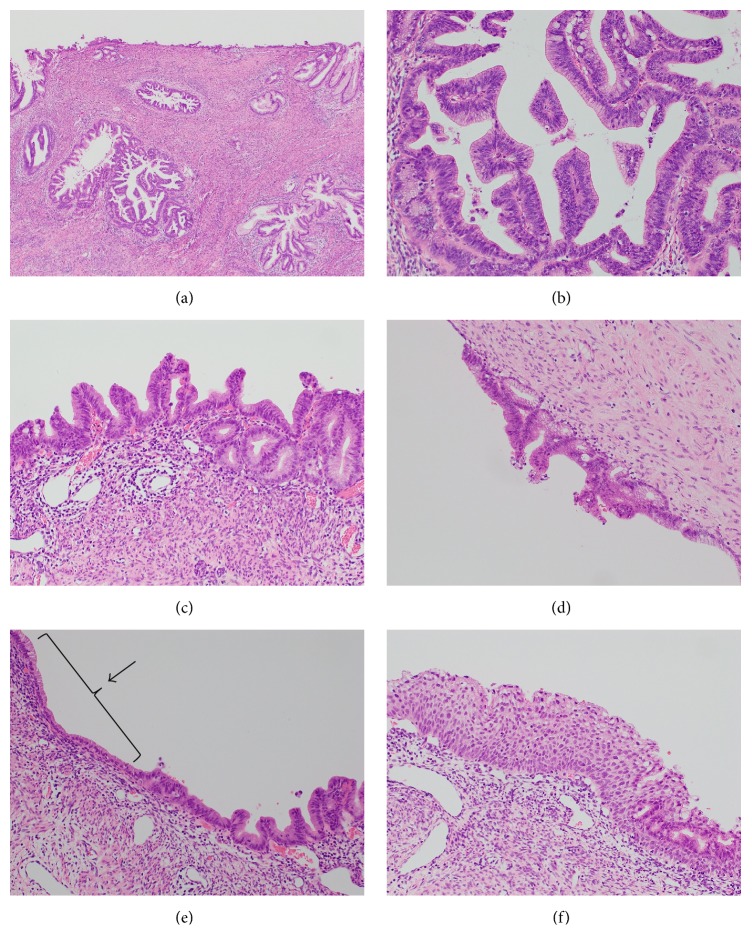
Intraepithelial lesions. (a) H-E stain. Endocervical lesions. Neoplastic epithelium replaces normal epithelium on the cervical surface and glands. (b) Neoplastic epithelium with atypical epithelial and glandular cells with nuclear irregularity, variable sizes, mitosis, and apoptosis. Some glands have abundant pale eosinophilic cytoplasm, and partial glands have intestinal differentiation with goblet cells. AIS. (c) H-E stain. Endometrial lesions. Similar lesions in the endocervical mucosa. Cytological atypia was less than Figures 4(a) and 4(b); however they have mild nuclear atypia and pseudostratification. (d) H-E stain. The left tube mucosa is abnormal with mild nuclear pseudostratification, hyperchromasia, and enlargement, and tumor cells had mitosis. However, dysplasia was less than AIS. (e) H-E stain. Endometrium without lobular architecture and with the characteristic “pale pink” cytoplasm that was next to AIS. Simple gastric metaplasia (as indicated by arrow). (f) H-E stain. The endometrium showed lesions of stratified mucin-producing intraepithelial lesion (SMILE) focally. The stratified epithelium contains cells with mucin vacuoles in all cell layers. Nuclear atypia and hyperchromasia figures are present.

**Figure 5:**
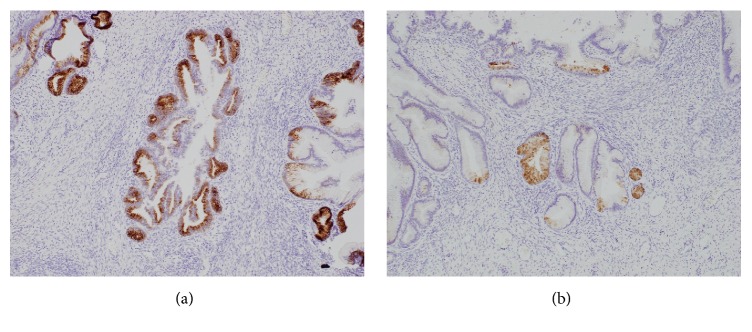
Immunohistochemical results. (a) MUC6 stain. Tumor cells were partially positive. (b) HIK1083 stain. Tumor cells were focally positive.

Furthermore, irregularity of the epithelium was noted in the cervical mucosa and endometrium. Histologically, atypical epithelial and glandular cells with nuclear irregularity, variable sizes, mitosis, and apoptosis and without stromal invasion were noted in the cervix. Part of the glands had abundant pale eosinophilic cytoplasm, and part of the glands had intestinal differentiation with goblet cells (Figures 4(a) and 4(b)). These findings led to the diagnosis of adenocarcinoma in situ (AIS). The carcinoma cells consisted mostly of gastric-type cells and partly of intestinal type cells from the histological features and immunohistochemical findings (Figures 5(a) and 5(b), Table 2). Similar intraepithelial carcinoma cells were present in the endometrium as continuous and skip patterns and in the left fallopian tube as a partial lesion. Stromal invasion was noted in all lesions (Figures 4(c) and 4(d)). In the cervix and endometrium, mucinous metaplasia and stratified mucinous-producing intraepithelial lesion (SMILE) was admixed with intraepithelial carcinoma lesions (Figures 4(e) and 4(f)). In the left tube, atypical epithelial cells that had less atypia compared with carcinoma were admixed.

## 3. Discussion

Synchronous gynecological cancers are rare [8–10]. From the detailed literature reviews of uterine sarcoma with synchronous occurrence of carcinoma as indicated in Table 3, the present case is the first case with synchronous occurrence of leiomyosarcoma and adenocarcinoma in situ in the cervix [3–7]. Concerning the diagnosis of leiomyosarcoma, three differential diagnoses were considered: leiomyosarcoma, inflammatory myofibroblastic tumor (because these lesions had lymphocytic infiltration), and carcinosarcoma (because focally spindle atypical cells and epithelial lesions were very close). We denied the possibility of an inflamed myofibroblastic tumor because the lesion had high-grade cellular atypia and high mitotic activity and was ALK negative, and we denied the possibility of carcinosarcoma as a result of the absence of a carcinomatous element within the sarcoma lesion. Leiomyosarcoma was finally diagnosed from histological appearances and positive H-caldesmon results, although desmin was negative.

However, in the present case, gastric-type AIS (gAIS) was noted in the cervix, endometrium, and tube. Most endocervical glandular malignancies and their precursors are associated with high-risk human papilloma virus (HPV); however, some endocervical glandular gastric-type lesions were not associated with HPV. In the 2014 World Health Organization (WHO) classification, malignancies and their precursors of gastric-type cervical lesions, including lobular endocervical glandular hyperplasia (LEGH), minimal deviation adenocarcinoma (MDA), and gastric-type adenocarcinoma (GAS), were listed as endocervical lesions with a gastric phenotype [11, 12]. In particular, GAS is defined as a neoplasm composed of cells with abundant pale or eosinophilic cytoplasm and distinct cell borders, and it is considered that a precursor of GAS is gAIS. gAIS is defined as preexisting endocervical glands that are replaced by atypical columnar cells with abundant pale to eosinophilic cytoplasm and distinct cell borders without lobular architecture. Immunohistochemically, the glandular cells are focally positive for MUC6 and/or HIK1083 and sometimes diffusely positive for p53. ER and PgR are negative, and p16 is usually negative [13]. The classification of gastric-type cervical lesions has been established; the cases previously diagnosed as cervical type mucinous adenocarcinoma may be included in GAS and gAIS; there is a high probability that case reports will increase in the future [14].

**Figure 6:**
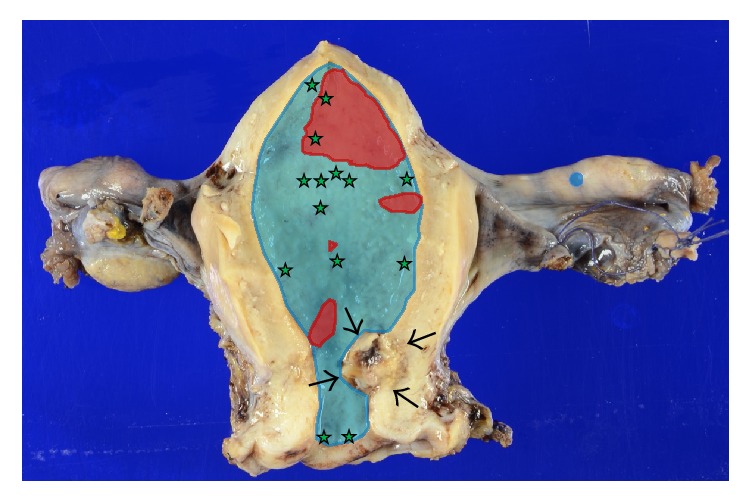
Distribution of epithelial lesions. Blue: gAIS. Red: simple gastric metaplasia. Green star: SMILE. The tubal lesion is a skip lesion. Cervical leiomyosarcoma is indicated by arrows.

Our case was diagnosed as endocervical gAIS from histological features and immunohistochemical results. In addition, synchronous occurrence of intraepithelial carcinoma was also noted in the endometrium with continuous and skip patterns and in the tube with partial lesions. Talia et al. reported nine cases in total; of these, three cases had intraepithelial spreading to endometrium [15]. We compared our case with these three cases of gAIS with intraepithelial spreading to the endometrium (there was extension to the lower segment, and, in two cases, there was involvement of the endometrium in the lower corpus with continuous lesions) (Table 4). Our case not only had continuous lesions to the endometrium but also had tubal skip lesions that were confirmed by continuous sectioning (Figure 6). However, because the tubal skip lesions were similar to other lesions, we suggested that these lesions were most likely not multifocal lesions but a series of lesions.

## 4. Conclusion

This is the first report of coincidental primary cervical leiomyosarcoma and cervical gastric-type AIS with intraepithelial spreading to the corpus and fallopian tube.

## Figures and Tables

**Table 1:** Immunohistochemical results of the endocervical tumor.

AE1/AE3	−
CAM5.2	−
34BE12	−
EMA	−
Vimentin	+
SMA	+
Desmin	−
H-caldesmon	+
CD10	−
ALK	−
Ki-67	10%

**Table 2:** Immunohistochemical analysis for each epithelial lesion.

Lesions	p53	p16	MUC2	MUC6	HIK1083	Ki-67	p40
AIS (endocervical)	Focal +	−	−	Partial+	Focal +	80%	ND
AIS (endometrium)	Focal +	−	−	Partial+	Focal +	70%	−
Simple gastric metaplasia	−	−	−	Partial+	Focal +	50%	−
SMILE	Focal +	−	−	−	−	40%	+
Tube	ND	ND	ND	Partial+	Focal +	40%	ND

ND, not done.

**Table 3:** Cases of synchronous primary malignant neoplasms: a summarizing review of the references.

case	Reference	Age	Location of leiomyosarcoma	Other cancers
(1)	Winston et al.	77	Uterine cervix	Squamous cell carcinoma of uterine cervix

(2)	Winston et al.	53	Uterine cervix	Squamous cell carcinoma of uterine cervix

(3)	Dudzik et al.	60	Uterine corpus	Endometrioid adenocarcinoma, G2 of uterine corpus

(4)	Kaite et al.	60	Uterine corpus	Endometrioid adenocarcinoma, G2 of uterine corpus, and bilateral serous cystadenofibromas

(5)	Isin et al.	56	Uterine corpus	Well-differentiated ovarian mucinous cystadenocarcinoma and endometrioid adenocarcinoma, G1 of uterine corpus

(6)	Sheyn et al.	66	Uterine corpus	Endometrioid adenocarcinoma, G1 of uterine corpus

(7)	Our case	66	Uterine cervix	Gastric type adenocarcinoma in situ of uterine cervix

TC, tunnel cluster; TZ, transformation zone; LEGH, lobular endocervical glandular hyperplasia; SGM, simple gastric metaplasia; SMILE, stratified mucin-producing intraepithelial lesion.

**Table 4:** Cases of gastric type AIS with intraepithelial spreading to the endometrium: a summarizing review of the references.

Case	Reference	Age	Location of AIS	Form of AIS type	Histological subtype of epithelial lesions	Other findings
(1)	Karen et al.	61	Proximal to TZ, lower uterine segment, and endometrium	Mixed gastric and intestinal type	None	None
(2)		73	TZ, lower uterine segment, and endometrium	Mixed gastric and intestinal type	SGM	None
(3)		52	TZ and lower uterine segment	Purely gastric type	TC, LEGH	None

(4)	Our case	66	Whole cervix, endometrium, and focally left tube	Mixed gastric and intestinal type	SMG, SMILE	Cervical leiomyosarcoma
